# Performance of Quantification of Modified Hodge Test: An Evaluation with *Klebsiella pneumoniae* Carbapenemase-Producing Enterobacteriaceae Isolates

**DOI:** 10.1155/2014/139305

**Published:** 2014-03-26

**Authors:** Vanessa Bley Ribeiro, Adriano Rostirolla Linhares, Alexandre P. Zavascki, Afonso Luis Barth

**Affiliations:** ^1^Faculdade de Farmácia, Universidade Federal do Rio Grande do Sul (UFRGS), 2752 Ipiranga Avenue, 90160-093 Porto Alegre, RS, Brazil; ^2^Laboratório de Pesquisa em Resistência Bacteriana (LABRESIS), Centro de Pesquisa Experimental, Hospital de Clínicas de Porto Alegre (HCPA), 2350 Ramiro Barcelos Street, 90035-903 Porto Alegre, RS, Brazil; ^3^Infectious Diseases Service, HCPA, 2350 Ramiro Barcelos Street, 90035-903 Porto Alegre, RS, Brazil

## Abstract

Modified Hodge Test (MHT) has been suggested as screening tests for carbapenemases, but concerns regarding its difficult interpretation and common false-positive results obtained in the presence of other **β**-lactamases have been noted. This study aimed to quantify the enhanced growth formed by the indicator strain and thus evaluate the performance of a quantitative interpretation of MHT for KPC screening. MHT was performed in 50 KPC-producing isolates and 334 non-carbapenemase-producing isolates, using ertapenem (ETP) and meropenem (MEM) as substrates. The size of enhanced growth of indicator strain was measured for each isolate tested and for the positive control used, and a ratio was calculated. Our results revealed 17 different ETP and MEM ratios, with distinct sensitivity (SN) and specificity (SP). Higher SN combined to higher SP was achieved when ETP and MEM ratios were 0.45, with a SN value of 96% for both substrates and SP values of 99.4% and 100% for ETP and MEM, respectively. The quantification with both substrates increased SP of the test for KPC detection. Considering that MHT is the unique phenotypic test that is referred to by CLSI, a more accurate approach for its interpretation could be applied to make it a more useful tool.

## 1. Introduction

The global spread of* Klebsiella pneumoniae* carbapenemases (KPC) in Enterobacteriaceae in the last decade poses as a serious public health threat, since it leads to variable levels of carbapenem resistance and few therapeutic options remain available for treating such infections [1, 2]. Moreover, KPC codifying genes are harbored in genetically mobile elements allowing their rapid spread among gram-negative rods [3].

Molecular methods are the gold standard to detect the KPC genes; however, although no longer recommended neither by CLSI nor by EUCAST, screening test may be necessary to discriminate which isolates with reduced susceptibility to carbapenems are more likely to produce KPC, particularly in low-income settings. Many screening tests for Ambler Class A carbapenemases have been presented in recent years, including the Modified Hodge Test (MHT) and tests based on boronic acid (BA) [4–6], as well as the combined use of *β*-lactamase inhibitors, including BA, EDTA, dipicolinic acid, and cloxacillin in order to differentiate Class A from other carbapenemases, such as metallo-*β*-lactamases and AmpC *β*-lactamases [7, 8]. Although MHT often presents high sensitivity (>90%) [4, 5, 9], its interpretation is usually difficult and also subjective [10, 11]. Moreover, many studies have demonstrated positive results in MHT in the presence of other *β*-lactamases, such as ESBL and AmpC coupled with porin loss [5, 10, 12, 13]. Although some new and more practical phenotypic methods have been proposed for carbapenemase detection [14, 15], MHT is inexpensive and accessible for virtually all clinical laboratories.

Since MHT positivity depends on the enzymatic activity, it should be expected that the enhanced growth intensity would be bigger with stronger hydrolytic activity of the enzyme. In this study, we evaluate the sensitivity (SN) and specificity (SP) of a quantitative interpretation of MHT for KPC detection.

## 2. Materials and Methods

### 2.1. Bacterial Isolates

A panel of distinct genera of Enterobacteriaceae resistant or with reduced susceptibility to ertapenem (ETP) and/or meropenem (MEM) by disc-diffusion test (zone of inhibition ≤ 21 mm) was included in the study [16]. A total of 50 KPC-producing and 334 non-carbapenemase-producing clinical isolates were recovered from 13 Brazilian hospitals from 2009 to 2011. All of them were previously identified by conventional techniques and by use of VITEK2 (bioMeriéux, France). CarbapenemsMICs were performed according to CLSI [17] and the distribution of both MIC_50_ and MIC_90_ groups is shown in Table 1. Carbapenemases were first investigated by multiplex real-time PCR for detection of *bla*_KPC_, *bla*_VIM_, *bla*_GES_, *bla*_NDM_, *bla*_OXA-48_, and *bla*_IMP_ genes [18]. Of the carbapenemases evaluated by PCR, only the KPC enzyme was detected among the isolates. All isolates were double-checked for the presence of *bla*_KPC_ by a different set of primers in a simplex PCR [19]. The positive control strains used in this study included a KPC-producing* K. pneumoniae *ATCC BAA-1705, a VIM-producing* P. aeruginosa*, a GES-producing* K. pneumoniae*, a NDM-producing* K. pneumoniae*, a OXA-48-producing* K. pneumoniae,* and a IMP-producing* P. aeruginosa.*

### 2.2. MHT Performance and Quantification

**Figure 1:**
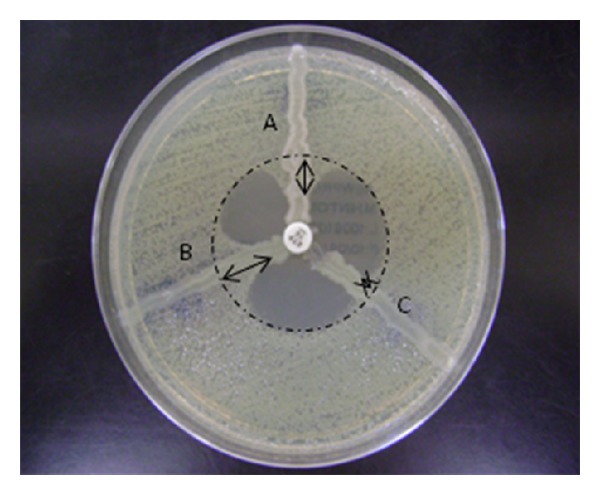
Quantification of Modified Hodge Test (MHT). The enhanced growth ratio of ETP and MEM subtracts were obtained from the measure (mm) of the enhanced growth of* E. coli* ATCC 25922 for both the isolate and the KPC-producing positive control (*K. pneumoniae* ATCC BAA 1705) and a ratio was calculated. In this example, B is the positive control and the enhanced growth measured for ETP was 10 mm. The enhanced growth measured for isolates A and C was 5 and 2 mm, respectively, resulting in ETP enhanced ratios of 0.5 and 0.2, respectively.

The MHT was performed for all isolates as recommended by CLSI [17]. In order to investigate the performance of both substrates, ETP and MEM disks were placed on the agar plate seeded with* E. coli* ATCC 25922 (indicator strain). The isolates were inoculated in a straight line out from the edge of the disk to the edge of the plate. The plates were incubated at 35 ± 2°C during 20 hours. The KPC-producing* K. pneumoniae* ATCC BAA 1705 was used as positive control in all experiments. For both substrates, the size of enhanced growth of indicator strain was measured in millimeters (mm) with a ruler, for each isolate tested and for the positive control used in each experiment separately, as demonstrated in Figure 1. For data analysis, a ratio was calculated according to the formula below, and then an ETP enhanced growth ratio and a MEM enhanced growth ratio were established for all isolates evaluated:

(1) R=(size  of  enhanced  growth  obtainedfrom  isolate  tested (mm))×(size  of  the  enhanced  growth  obtainedfrom  positive  control  strain (mm))−1.

The use of a ratio instead of using only the measurement of the enhanced growth generated by each isolate was chosen in order to minimize the variations inherent to the method, since it minimizes possible distinct growths that a same isolate could produce in a distinct day test and also discounts variations due to different agar media or disk suppliers, for example. The readings were done through the back of the plate in a dark background. To ensure the test reproducibility and the intra- and interobserver integrity, 30 isolates (15 KPC-producers and 15 KPC-nonproducers) were randomly selected to be tested again and the interpretation was performed by two independent investigators who were blinded for the carbapenemase results.

### 2.3. Statistical Analysis

The SN, SP, and receiver operating characteristic (ROC) curves and their respective 95% CI for ETP and MEM ratios were calculated using SPSS version 18.0. SN and SP were calculated using PCR as gold standard. The positive predicted values (PPV) and negative predictive values (NPV) of the test using both substrates were calculated according to estimated population prevalence of *bla*_KPC-2_. Agreement between intraobserver of two distinct investigators and interobserver readings was assessed by Bland-Altmann analysis, assuming the first reading as standard. Statistical significance was considered when the *P* value was <0.05.

## 3. Results

All isolates were submitted to MHT quantification and revealed 17 different ETP and MEM ratios by the ROC curves. The SN and SP of conventional reading of MHT (any enhanced growth, represented by lower ratios), with both ETP and MEM as substrates, were 100% and 28.4%. Depending on the enhanced growth ratio used for positive or negative MHT result, the SN and SP of ETP for KPC detection ranged from 58% to 100% and from 28.4% to 99.7%, respectively (Table 2), and from 54% to 100% and 28.4% to 100% for MEM, respectively (Table 3). As both ETP and MEM ratios increase, the SP also increases. In contrast, as both increase, the SN decreases.

The highest combined SN and SP values obtained for both substrates were a ratio = 0.45 (Tables 2 and 3). If ratios <0.45 were assigned as a positive MHT, two KPC-producing isolates would be considered negative for both substrates: an* E. cloacae *(ETP ratio = 0.2 and MEM ratio = 0.3) and a* S. marcescens* (ETP and MEM ratio = 0.2). Among non-carbapenemase-producing isolates, two* E. cloacae *showed a ratio ≥ 0.45 only when ETP was used as substrate, with ratios of 0.5 and 1.0. For these two isolates, ESBL production was performed by phenotypic tests using combination-disk assay with clavulanic acid as inhibitors, as well as by PCR for the presence of *bla*_CTX-M_ [17, 20]. The presence of AmpC was also investigated, as described by Giske et al. [8]. Both isolates presented positive results with cloxacillin assay, indicating the presence of an AmpC, and the first one also showed the presence of a CTX-M.

**Figure 2:**
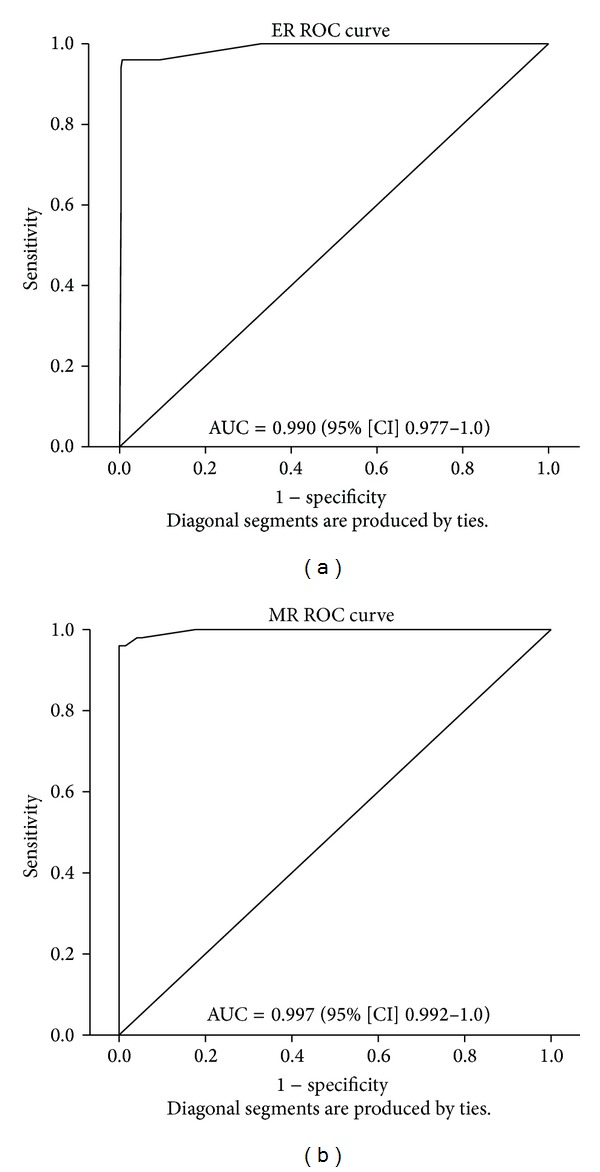
Ertapenem enhanced growth ratio (ER) ROC curve (a) and meropenem enhanced growth ratio (MR) ROC curve (b). AUC = area under the curve; CI = confidence interval.

The PPV and NPV for quantitative MHT are shown in Table 4. The area under the curve (AUC) calculated for ER was 0.990 (95% confidence interval [CI] 0.977–1.0) (Figure 2(a)) and for MR 0.997 (95% [CI] 0.992–1.0) (Figure 2(b)). The Bland-Altmann analysis indicated that there was intra- and interobserver agreement in the readings. The comparisons included intraobserver agreement of ER for two different investigators (*P* = 0.081 and *P* = 0.073), intraobserver agreement of MR also for both investigators (*P* = 0.962 and *P* = 0.078), and interobserver agreement for both substrates (*P* = 0.894 and *P* = 0.136). Most reading disagreements were ≤1 mm.

## 4. Discussion

KPC dissemination among Enterobacteriaceae isolates in recent years has challenged clinical laboratories to provide a rapid, inexpensive, and accurate result of which isolate with reduced susceptibility to carbapenems would more likely produce these enzymes. Although MHT fulfilled the first two characteristics, it is an inaccurate test, since false-positive results may be found in isolates producing other *β*-lactamases with some marginal carbapenemase activity, such as AmpC and ESBLs [5, 10].

The results of the present study indicate that both SN and SP vary according to the size of the enhanced growth of the indicator strain. For both substrates analyzed, ETP and MEM, our results clearly demonstrated that higher ratios originated higher SP values, whereas lower ratios originated higher SN. Therefore, as previously hypothesized, ETP and MEM ratios are directly proportional to SP and inversely proportional to SN in carbapenemase detection. It is interesting to note that, using the conventional qualitative MHT interpretation, the SN was 100% for both substrates; however, unacceptable low SP values (28.4%) were obtained. In our study, the SN of ETP and MEM ratios ≥0.45 (96%) was only slightly decreased in comparison with the SN (100%) of the conventional qualitative MHT reading, and it was similar to the SN values of MHT reported in other studies (>90%) [17, 21, 22]. The PPV and NPV found for both substrates indicated that the quantitative MHT is a useful and reliable test to be used in settings with low and high prevalence of KPC-2-producing bacteria. Regarding overall accuracy, MEM tended to have a better performance compared to ETP, with an AUC of 0.997 compared to 0.990 for the last. These results were driven by the better SP values when using MEM as substrate. The agreement analysis of intra- and interobserver indicated that the readings are reproducible and that the ratios generated on different days are reliable.

A limitation of our study was that only KPC carbapenemase was assessed, and these findings may not be applied to other carbapenemases types. Considering that MHT is not specific for KPC detection, it could be expected that the SN and SP would vary in a similar way with other carbapenemases, although further studies are needed to confirm this hypothesis. Quantification of MHT primarily aims to differentiate the presence of carbapenemase production from other enzymes with minimal carbapenemase activity. It is also known that the 0.45 cut-off must not be taken as definitive and its accuracy should be further investigated.

## 5. Conclusions

Our study showed that distinct sizes of enhanced growth of the indicator strain in MHT determine different SN and SP for the detection of carbapenemase-producing isolates. The quantification of MHT with either ETP or MEM as substrates increases SP of the test for the detection of carbapenemase activity in Enterobacteriaceae with minimal impairment in the SN, when compared with the conventional reading of the MHT. Although other phenotypic tests are currently available, the quantification of MHT may be an alternative screening test, particularly for clinical laboratories from low-income regions. The quantification of MHT must be further investigated with other non-KPC-type carbapenemases.

## Figures and Tables

**Table 1:** Minimal inhibitory concentration (MIC) of carbapenems of the 384 isolates evaluated.

Isolates	*n*					MIC (mg/L)^a^							
		Imipenem				Meropenem				Ertapenem			
		MIC^b^	MIC_50_	MIC_90_	Range	MIC^b^	MIC_50_	MIC_90_	Range	MIC^b^	MIC_50_	MIC_90_	Range
KPC-producing isolates	50	—	16	128	1.0–256	—	32	128	4.0–>256	—	64	256	4.0–>256
*Klebsiella* spp.	36	—	16	64		—	32	64		—	64	128	
*Enterobacter* spp.	10	—	8	64		—	16	128		—	32	256	
*S. marcescens *	3	—	128	256		—	32	64		—	64	128	
*K. georgiana *	1	256	—	—		128	—	—		128	—	—	
Non-carbapenemase-producing isolates	334	—	0.5	2.0	≤0.5–32		0.5	4.0	≤0.5–32		4.0	16	≤0.5–128
*Enterobacter* spp.	220	—	0.5	2.0			0.5	2.0			4.0	8.0	
*Klebsiella *spp.	89	—	1.0	4.0			1.0	8			4.0	32	
*E. coli *	17	—	0.5	4.0			1.0	4.0			4.0	16	
*S. marcescens *	5	—	1.0	4.0			1.0	4.0			2.0	8.0	
*P. mirabilis *	1	2.0	—	—		0.5	—	—		0.5	—	—	
*M. morganii *	1	4.0	—	—		0.5	—	—		0.5	—	—	
*C. freundii *	1	≤0.5	—	—		0.5	—	—		0.5	—	—	

^a^Determined by broth microdilution [17].

^b^Result of MIC of species with a single isolate.

**Table 2:** Sensitivity (SN) and specificity (SP) of distinct values of ertapenem (ETP) enhanced growth ratios.

ETP ratios	SN (%)	SP (%)
0.045	100.0	28.4
0.095	100.0	29.3
0.106	100.0	63.5
0.118	100.0	64.1
0.134	100.0	65.6
0.171	100.0	67.1
0.211	96.0	90.7
0.254	96.0	91.3
0.293	96.0	92.5
0.317	96.0	97.0
0.367	96.0	97.3
0.450	96.0	99.4
0.583	94.0	99.7
0.683	92.0	99.7
0.750	82.0	99.7
0.850	70.0	99.7
0.950	58.0	99.7

**Table 3:** Sensitivity (SN) and specificity (SP) of distinct values of meropenem (MEM) enhanced growth ratios.

MEM ratios	SN (%)	SP (%)
0.045	100.0	28.4
0.095	100.0	29.3
0.113	100.0	79.0
0.134	100.0	79.9
0.162	100.0	81.7
0.191	100.0	82.3
0.225	98.0	94.6
0.268	98.0	95.2
0.293	98.0	95.8
0.350	96.0	98.5
0.450	96.0	100.0
0.550	94.0	100.0
0.560	90.0	100.0
0.750	86.0	100.0
0.838	66.0	100.0
0.888	62.0	100.0
0.950	54.0	100.0

**Table 4:** Positive predictive values (PPV) and negative predictive values (NPV) of the quantitative Modified Hodge Test, according to estimated population prevalence of *bla*_KPC-2_.

Population prevalence of *bla*_KPC-2_	MEM		ERT	
	PPV (95% CI)	NPV (95% CI)	PPV (95% CI)	NPV (95% CI)
10%	97.3% (87.8–99.4%)	99.6% (98.5–99.9%)	94.7% (83.8–98.4%)	99.6% (98.5–99.9%)
20%	98.8% (94.2–99.8%)	99.0% (96.7–99.7%)	97.6% (92.1–99.3%)	99.0% (96.7–99.7%)
30%	99.3% (96.5–99.9%)	98.3% (94.5–99.5%)	98.6% (95.2–99.6%)	98.3% (94.5–99.5%)
50%	99.7% (98.0–99.9%)	96.1% (88.0–98.8%)	99.4% (97.9–99.8%)	96.1% (88.0–98.3%)
70%	99.9% (99.3–100%)	91.4% (75.9–97.3%)	99.7% (99.1–99.9%)	91.4% (75.9–97.3%)

MEM: meropenem; ERT: ertapenem.
